# *Bacillus halotolerans* Strain 105 enhances wheat growth and suppresses diverse phytopathogens under saline conditions: an integrated genomic and phenotypic study

**DOI:** 10.3389/fmicb.2026.1812027

**Published:** 2026-04-22

**Authors:** Hu Liu, Yuyun Gao, Hongmin Liu, Kaili Wang, Fangying Wang, Yu Zhang, Zhitong Jiang, Qingmin Chen, Jun Wang

**Affiliations:** College of Food Science and Engineering, Shandong Agriculture and Engineering University, Jinan, China

**Keywords:** *Bacillus halotolerans*, biological control, indole-3-acetic acid, plant growth-promoting rhizobacterium, rhizosphere colonization, soil salinity

## Abstract

Soil salinization and phytopathogen infection threaten global crop productivity. Plant growth-promoting rhizobacteria that alleviate abiotic stress while suppressing disease offer a sustainable solution. We aimed to identify a strain with robust salt tolerance, broad-spectrum antagonism, and effective root colonization. A novel halotolerant bacterium isolated from plant rhizosphere at an artificial lakeside was evaluated for plant growth-promoting and biocontrol capacities through phenotypic assays. High-quality whole genome sequencing and bioinformatic analysis were used for functional annotation. The results showed that Strain 105, identified as *Bacillus halotolerans*, exhibited exceptional tolerance to NaCl concentrations up to 14% (w/v). Under 200 mmol/L NaCl stress, inoculation significantly enhanced wheat seedling growth, increasing root length by 20% and plant height by 18% compared to controls. Strain 105 displayed strong *in vitro* antagonistic activity against four phytopathogenic fungi that infect crops during the growing period, three postharvest phytopathogenic fungi, and three pathogenic bacteria. Genomic analysis revealed a 4.2-Mb chromosome encoding four key functional modules: (i) osmoprotective systems for glycine betaine and proline metabolism; (ii) an indole-3-pyruvate pathway for indole-3-acetic acid biosynthesis; (iii) multiple secondary metabolite biosynthetic gene clusters (e.g., iturin, fengycin, bacillibactin, and bacilysin); and (iv) a complete set of genes for chemotaxis, biofilm formation, and root adhesion. In conclusion, the multifunctionality of *B. halotolerans* Strain 105 arises from the synergistic interplay of genetic modules, enabling it to thrive in saline conditions while promoting plant growth and suppressing diverse pathogens. Strain 105 is a promising candidate for developing next-generation microbial inoculants for sustainable agriculture in saline-alkali soils.

## 1 Introduction

Under the dual pressures of global climate change and arable land degradation, the sustainable development of agriculture is facing severe challenges. In particular, the damage caused by pathogenic microorganisms and the constraints imposed by saline-alkali stress have emerged as two critical factors limiting the safe production of grain crops.

On the one hand, systemic diseases induced by *Fusarium* spp.—such as vascular wilt caused by *F. oxysporum*, ear rot of maize triggered by *F. proliferatum* (Ghanbarzadeh et al., 2014), and various root and ear rot diseases in diverse crops— contribute to more than 50% yield loss, while the accompanying mycotoxins (e.g., zearalenone) pose a persistent threat to food security and public health (Dean et al., 2012). On the other hand, soft rot diseases caused by pathogenic bacteria including those of the genera *Pectobacterium* and *Dickeya* result in enormous economic losses throughout both the crop growth cycle and the postharvest supply chain (Tan et al., 2022; Yi et al., 2024). Moreover, soil salinization has affected over 30% of the world's arable land. By disrupting osmotic balance, inducing ionic toxicity and oxidative damage, soil salinization severely inhibits plant physiological metabolism and consequently reduces the yields of major food crops (Du et al., 2023; Osman, 2018). Faced with the complex challenges arising from the interplay of biotic and abiotic stresses, the development of synergistic green prevention and control strategies integrated with plant growth promotion approaches has become an urgent imperative for safeguarding global food security.

At present, chemical pesticide control and physical/chemical soil amelioration remain the primary strategies to address these challenges (Du et al., 2023). Nevertheless, the problems arising from these measures, including environmental pollution, ecological imbalance, and exorbitant application costs, have become increasingly prominent, necessitating the development of green, high-efficiency, and sustainable alternative strategies. Against this backdrop, plant growth-promoting rhizobacteria (PGPR) exhibit immense potential as an eco-friendly and sustainable biological solution. PGPR exert beneficial effects on plant health and growth through multiple mechanisms: (1) direct plant growth promotion, such as biological nitrogen fixation, phosphate solubilization, and biosynthesis of phytohormones including auxins; (2) indirect biological control, such as the production of antimicrobial secondary metabolites (e.g., lipopeptides, polyketides), ecological niche competition (e.g., siderophore secretion), and induction of plant systemic resistance; and (3) enhancement of abiotic stress tolerance, such as regulation of plant endogenous stress signaling pathways and promotion of osmoprotectant accumulation (Choudhary et al., 2025; Kumar et al., 2024; Nagrale et al., 2023). Therefore, the screening and identification of PGPR strains with multiple synergistic functions can help to realize the transition to green agriculture.

Among various PGPR genera, *Bacillus* has shown great application potential in agriculture owing to its strong environmental adaptability and functional diversity (Feng et al., 2024; Yuan et al., 2022). In particular, *Bacillus halotolerans* holds unique value for the microbial remediation of salinized soils, as it inherently possesses the ability to survive and metabolize in hyperosmotic environments. Studies have demonstrated that different strains of *B. halotolerans* exhibit either salt-tolerant growth-promoting activity (Wu et al., 2022) or antagonistic activity against specific pathogens (Kang et al., 2023; Madushani et al., 2025). Despite this promising potential in biological control and salt-tolerant plant growth promotion, there is a lack of multifunctional *B. halotolerans* strains capable of simultaneously exerting broad-spectrum antifungal/bacterial activity and significantly promoting wheat growth under high-salt conditions (e.g., 200 mmol/L NaCl). More importantly, the genetic basis underlying these synergistic biological functions remains to be systematically elucidated.

Although several halotolerant PGPR have been reported, few strains integrate all key characteristics of extreme salt tolerance (>1 mol/L NaCl), broad-spectrum antifungal activity, and indole-3-acetic acid (IAA)-mediated growth promotion. In addition, the complete genome sequence for such strains is lacking, which is needed to further elucidate the genetic basis of these traits. To address this gap, we isolated a novel *Bacillus halotolerans* strain, designated Strain 105, from a stressed soil environment. Integrating comprehensive phenotypic assays with high-quality whole genome sequencing, we characterized its functional capacity to (i) withstand extreme salinity, (ii) suppress diverse phytopathogens, and (iii) promote wheat growth under salt stress. Concurrently, we systematically mined its genome for key functional modules governing secondary metabolism, osmoadaptation, IAA biosynthesis, and rhizosphere colonization. This work provides both mechanistic insights and a promising microbial resource for developing next-generation bioinoculants tailored to saline–alkali soils.

## 2 Materials and methods

### 2.1 Test materials

Strain 105 was isolated in 2023 from the rhizosphere soil of Ilex chinensis near an artificial lake on the campus of Shandong Agriculture and Engineering University (Zibo, Shandong Province, China; GPS coordinates: 36°50′N, 117°55′E) was preserved in the Plant Molecular Biology Laboratory of Shandong Agriculture and Engineering University. Wheat (*Triticum aestivum* L.) was used as the host plant in this study. The cultivar used was “Jimai 22,” originated from the Shandong Academy of Agricultural Sciences, and the seeds were harvested in 2023. Pathogens (such as *F. oxysporum, F. moniliforme, F. solani, F. proliferatum, F. fujikuroi, Mucor circinelloides, Alternaria alternata, Escherichia coli, Staphylococcus aureus*, and *Pectobacterium brasiliensis*) were preserved in the Plant Molecular Biology Laboratory of Shandong Agriculture and Engineering University.

### 2.2 Biological characteristics of Strain 105

#### 2.2.1 Colony morphology observation

A single colony of Strain 105 was picked and inoculated onto Luria–Bertani (LB) solid medium (Qingdao Hopebio Biotechnology Co., Ltd, Qingdao, China) using the three-streak plate method. The inoculated plates were incubated at 37 °C for 24 h, after which the colony morphology was observed and recorded.

#### 2.2.2 Cell morphology observation

Cells in the logarithmic growth phase were collected for Gram staining, which was performed using a commercial Gram staining kit purchased from Qingdao Haibo Biotechnology Co., Ltd. (Qingdao, China).

### 2.3 Determination of plant growth-promoting traits of Strain 105

#### 2.3.1 Antimicrobial spectrum detection

Strain 105 was activated on LB medium cultured at 37 °C for 24 h. Pathogenic fungi (*F. oxysporum* CFP10, *F. moniliforme* CFP11, *F. solani* CFP13, *F. proliferatum* CFP12, *F. fujikuroi* L1, *Mucor circinelloides* PP3, *Alternaria alternata* TMP1) were activated on a potato dextrose agar (PDA) medium (Qingdao Hopebio Biotechnology Co., Ltd., Qingdao, China) with culture at 28 °C for 4 days. Antagonistic tests were carried out according to the method of Liu et al. (2020). In brief, an ager with activated fungi was placed on the center of the PDA plate and cultured at 28 °C for 2 days, followed by inoculation of activated Strain 105 around the fungi with culture proceeding at 28 °C for another 4 days. The presence of bacteriostatic bands between the fungi and Strain 105 indicated that Strain 105 exhibits antagonism against pathogenic fungi. We used *Escherichia coli* DH5-Alpha, *Staphylococcus aureus* BYJ1 (pathogenic bacteria), and *Pectobacterium brasiliensis* BY2 (the causal agent of pepper soft rot) as indicator strains, and adopted the Oxford cup method to assess their antagonistic activities.

#### 2.3.2 Detection of protease production ability of Strain 105

Strain 105 was inoculated on a medium plate of skim milk powder [Prepared using skim milk powder from Becton, Dickinson and Company, (Sparks, Maryland, USA) mixed with agar from Oxoid] and cultured at 37 °C for 2 days until a transparent circle formed around the colonies (Wang et al., 2020). The radio of the transparent circle diameter (*D*) to the colony diameter (*d*) was used for estimating the strain's ability to degrade protein.

#### 2.3.3 Detection of IAA secretion ability of Strain 105

The auxin secretion ability of Strain 105 was determined using the Salkowski colorimetric solution method, as described by Gordon and Weber (1951).

#### 2.3.4 Salt tolerance test of Strain 105

LB media supplemented with different concentrations of sodium chloride (NaCl; 8%, 10%, 12%, 14%) were prepared. The activated Strain 105 was inoculated into the LB liquid medium at an inoculum size of 1% (volume/volume). The inoculated cultures were then incubated in a constant-temperature shaking incubator at 37 °C with shaking at 180 rpm. Samples were taken every 2 h to determine the optical density at 600 nm (OD_600_) over a continuous 36-h period. Three replicates were established for each treatment group. A growth curve was plotted based on the OD_600_ values obtained.

#### 2.3.5 Growth-promoting experiment of Strain 105 on wheat under salt stress

Wheat seeds were surface-sterilized by soaking in 75% ethanol for 1 min, followed by immersion in 2% sodium hypochlorite (NaClO) solution for 5 min, and finally rinsed five times with sterile distilled water. Wheat seeds were soaked in distilled water for 1 h to ensure full water absorption. The seeds were evenly spread on moistened filter paper lining the bottom of a Petri dish. The Petri dishes were sealed with plastic wrap, and small holes were pricked into the wrap with toothpicks to allow air permeability. The seeds were cultured overnight in the dark at room temperature until radicle emergence.

The bacterial suspension was inoculated into LB liquid medium and incubated in a shaker at 37 °C for approximately 10 h until the bacterial concentration reached ~108 colony-forming units (CFU)/mL. Each bacterial suspension was diluted 50-fold for subsequent use, with the 50-fold diluted LB liquid medium serving as the control group.

Germinated wheat seeds were gently picked up with tweezers; seeds in the control group were dipped into the 50-fold diluted LB liquid medium, while those in the treatment groups were dipped into the respective 50-fold diluted bacterial suspensions. Subsequently, the seeds were placed on a thin layer of absorbent cotton at the bottom of hydroponic baskets containing half-strength Hoagland's nutrient solution supplemented with 200 mmol/L NaCl. There were 30 seeds per basket and five replicate baskets per group.

All hydroponic baskets were cultured at room temperature under light conditions with good ventilation. The wheat seedlings in each group were photographed to measure and record the growth status every week.

### 2.4 Genome sequencing and functional gene analysis of Strain 105

To understand the molecular mechanisms by which Strain 105 promotes plant growth, the complete genome sequence of Strain 105 was obtained using Illumina NovaSeq and PacBio platforms.

To determine the taxonomic status of Strain 105, multiple-sequence alignment of the 16S rRNA-encoding sequence was performed using MAFFT software, followed by phylogenetic tree construction with FastTree (Price et al., 2010). Average nucleotide identity (ANI) analysis of the chromosomal sequences was conducted on the IPGA v1.09 platform (Liu et al., 2022), and digital DNA–DNA hybridization (dDDH) was carried out using the genome-to-genome distance calculator (GGDC) 3.0 platform (Meier-Kolthoff et al., 2022). In addition, we searched for 20 closely related species in the database using FastANI software, and constructed a phylogenetic tree based on the core genes of the samples with UBCG software (Jain et al., 2018). The genome was annotated with the NCBI prokaryotic genome annotation pipeline (PGAP; http://www.ncbi.nlm.nih.gov/genome/annotation_prok/). The Cluster of Orthologous Groups (COG) of proteins annotations of protein-coding genes were performed with eggNOG-mapper software (Cantalapiedra et al., 2021). Secondary metabolism gene clusters in Strain 105 were predicted through the antiSMASH database (Version 8.0) (Blin et al., 2025).

### 2.5 Statistical analysis

The statistical significance of agronomic characters between groups were determined with *t*-tests in GraphPad Prism 7 (GraphPad Software, San Diego, CA, USA), with a significance threshold of *P* < 0.05. Graphs were drawn using GraphPad Prism 7.

## 3 Results

### 3.1 Morphological characteristics and taxonomic identification

**Figure 1:**
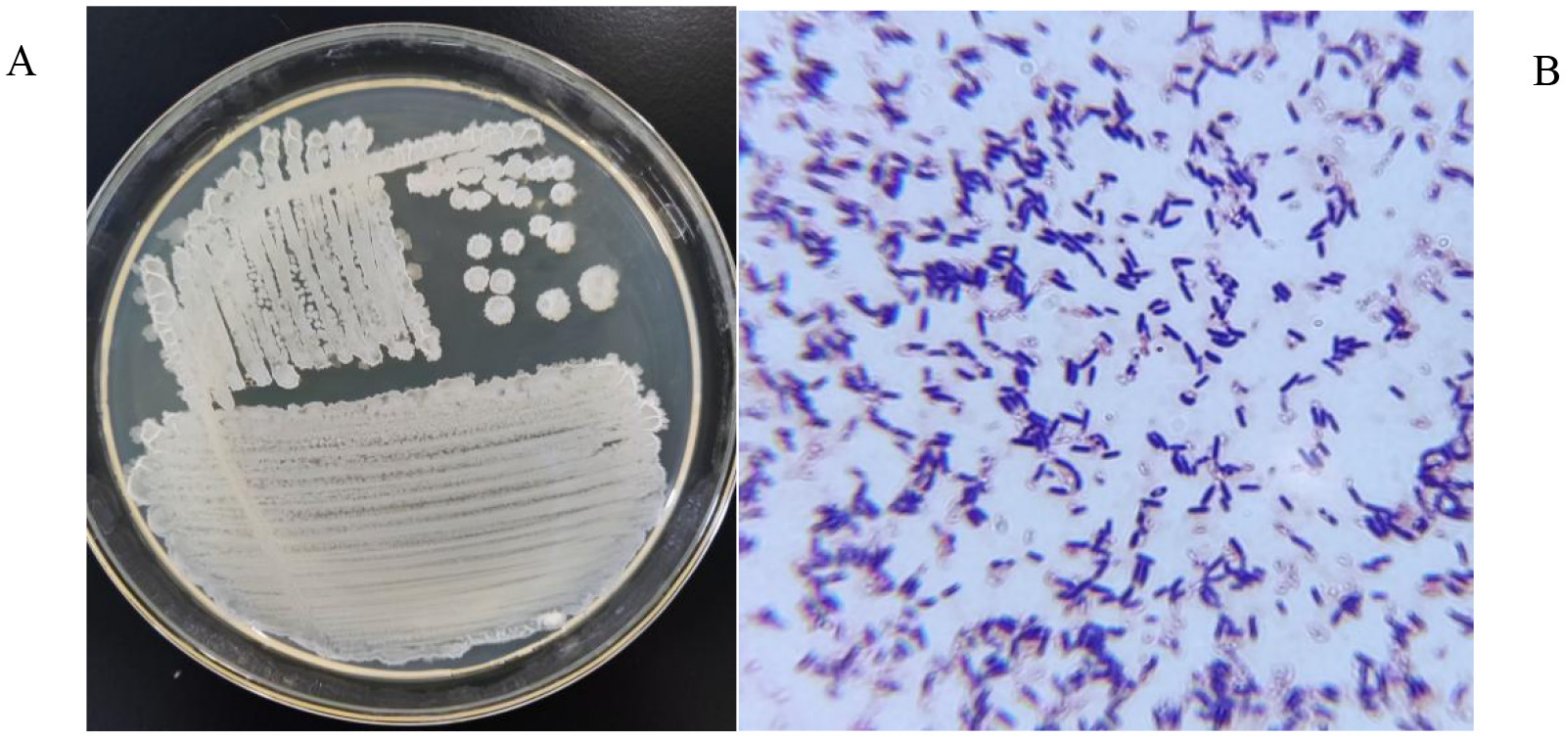
Morphological characteristics of Strain 105. **(A)** Colony morphology was observed on LB agar after incubation at 37 °C for 24 h. **(B)** Cell morphology was visualized using Gram staining (crystal violet/iodine/safranin) under a light microscope.

After 24 h of incubation on LB solid medium, Strain 105 formed white, wrinkled-surfaced colonies with relatively regular margins. These colonies were convex and difficult to pick with an inoculation loop (Figure 1A). Gram staining revealed that Strain 105 exhibited a rod-shaped morphology and was stained dark purple. Therefore, Strain 105 was identified as a Gram-positive bacterium (Figure 1B).

### 3.2 Functional phenotypic validation of Strain 105

#### 3.2.1 Broad-spectrum antagonistic activity of Strain 105

**Figure 2:**
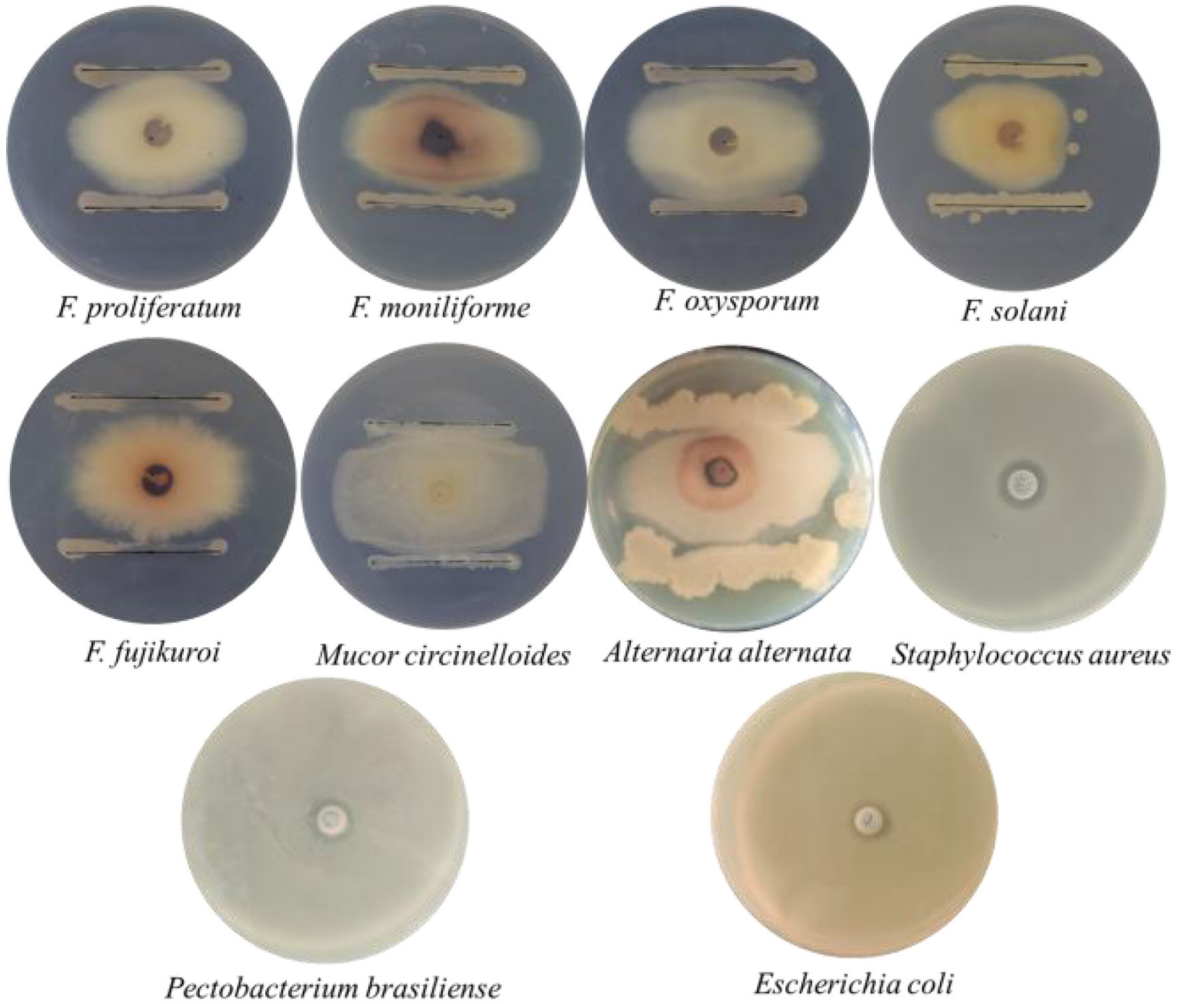
Antimicrobial spectrum of Strain 105.

Strain 105 exhibited inhibitory activity against a variety of phytopathogens *in vitro* (Figure 2). Importantly, the strain showed significant inhibitory effects on four phytopathogenic fungi that infect crops during the growing period, including *Fusarium oxysporum, Fusarium moniliforme, Fusarium solani*, and *Fusarium proliferatum*. Strain 105 also exerted notable suppression on three postharvest phytopathogenic fungi (i.e., *Fusarium fujikuroi, Mucor circinelloides*, and *Alternaria alternata*). Additionally, this strain displayed antagonistic activity against both pathogenic bacteria of humans (*Escherichia coli* and *Staphylococcus aureus*) and a phytopathogenic bacterium responsible for crop bacterial diseases (*Pectobacterium brasiliense*).

#### 3.2.2 Enzyme production and phytohormone biosynthesis capabilities

**Figure 3:**
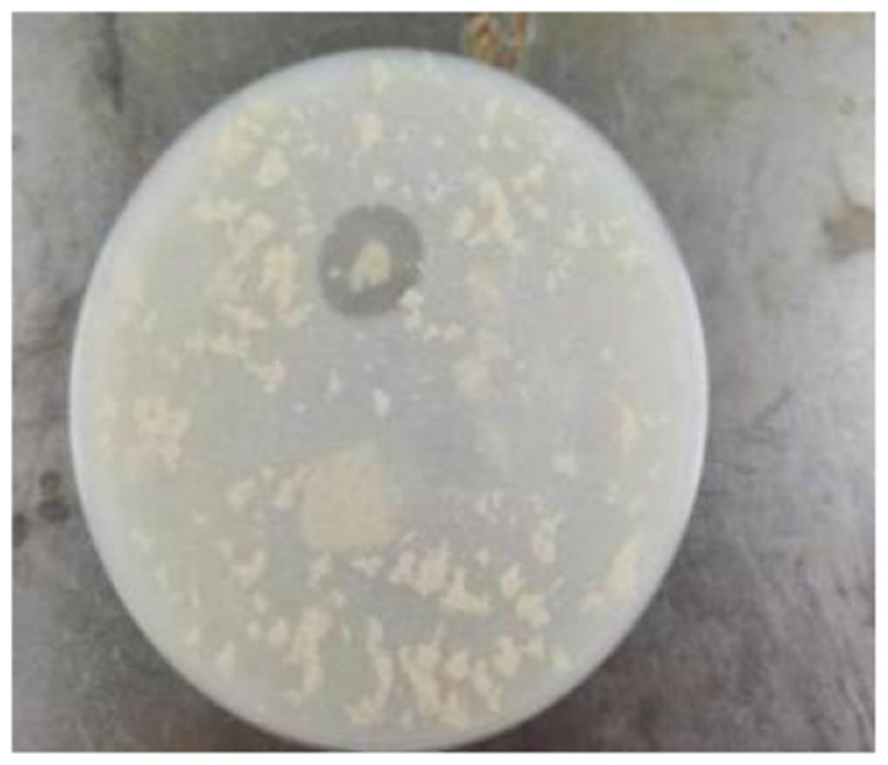
Protease production of Strain 105.

After Strain 105 was inoculated onto skim milk powder medium and incubated for 24 h, a distinct hydrolysis zone was observed surrounding the colonies (Figure 3). Quantitative determination demonstrated that the protease activity of this strain reached 216.42 IU.

The IAA secretion capacity was detected via the Salkowski colorimetric method. When Strain 105 was cultured in Landy liquid medium supplemented with l-tryptophan for 72 h, the IAA yield reached 13.6 μg/mL at an OD_600_ of 1.

#### 3.2.3 Extreme salt tolerance characteristics

**Figure 4:**
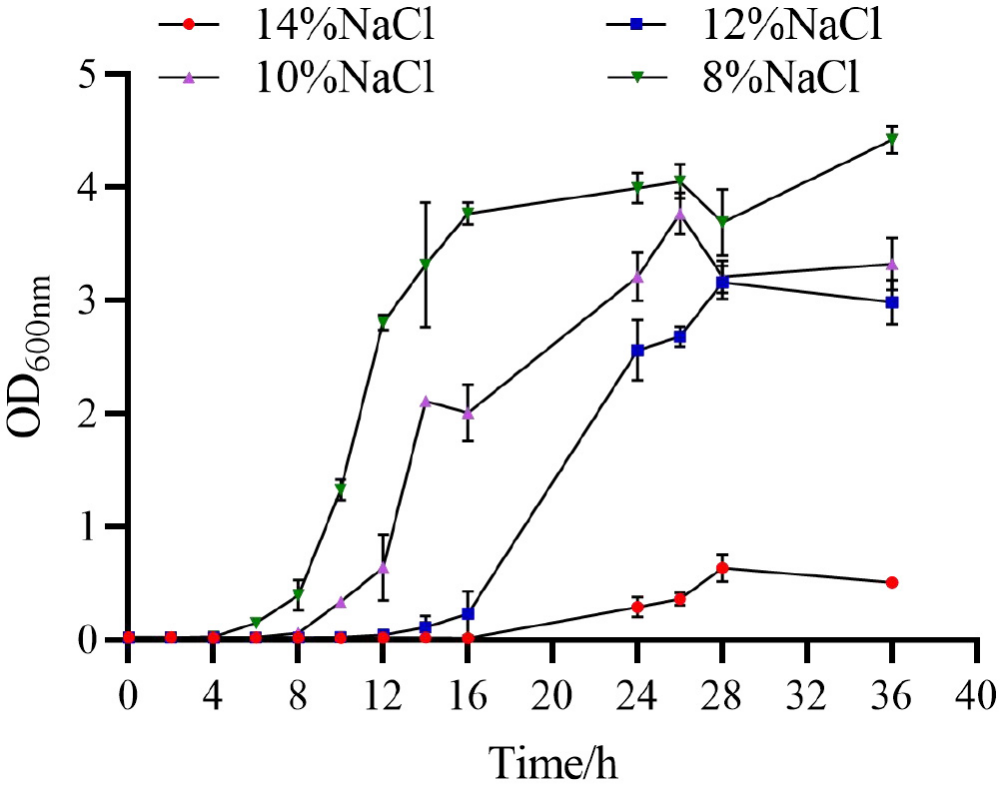
Effects of different NaCl concentrations on the growth of Strain 105.

Strain 105 exhibited exceptional tolerance to high salinity in liquid culture. Growth curves under different salt concentrations indicated that Strain 105 was able to complete its entire growth cycle across the NaCl concentration range of 8%−14% (Figure 4).

The 8% NaCl treatment group initiated growth at the earliest time point, entering the logarithmic growth phase at 8 h, and showed the optimal proliferation activity with the maximum OD_600_ value reaching 4.05. The 10% and 12% NaCl treatment groups entered the logarithmic growth phase at 8 h and 12 h, respectively, with maximum OD_600_ values of 3.76 and 3.16. Even under the stress of 14% NaCl, although Strain 105 presented a relatively long lag phase of 16 h, it still exhibited measurable growth, with a final reduction in biomass (OD_600_ = 0.636).

#### 3.2.4 Growth-promoting effects of Strain 105 on wheat under salt stress

**Figure 5:**
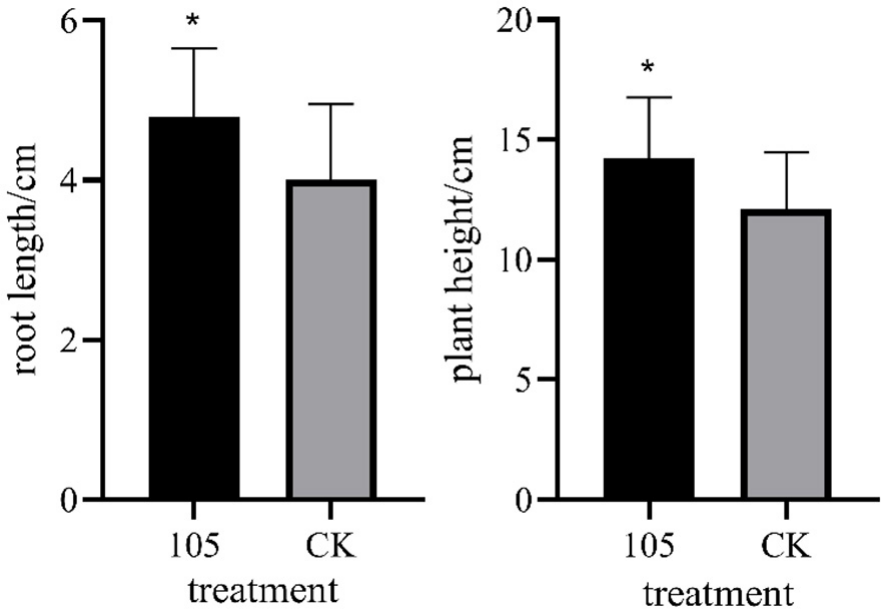
Effects of Strain 105 on plant height and root length of wheat under 200 mmol/L NaCl stress. * indicates significant difference at the *p* < 0.5 level.

**Figure 6:**
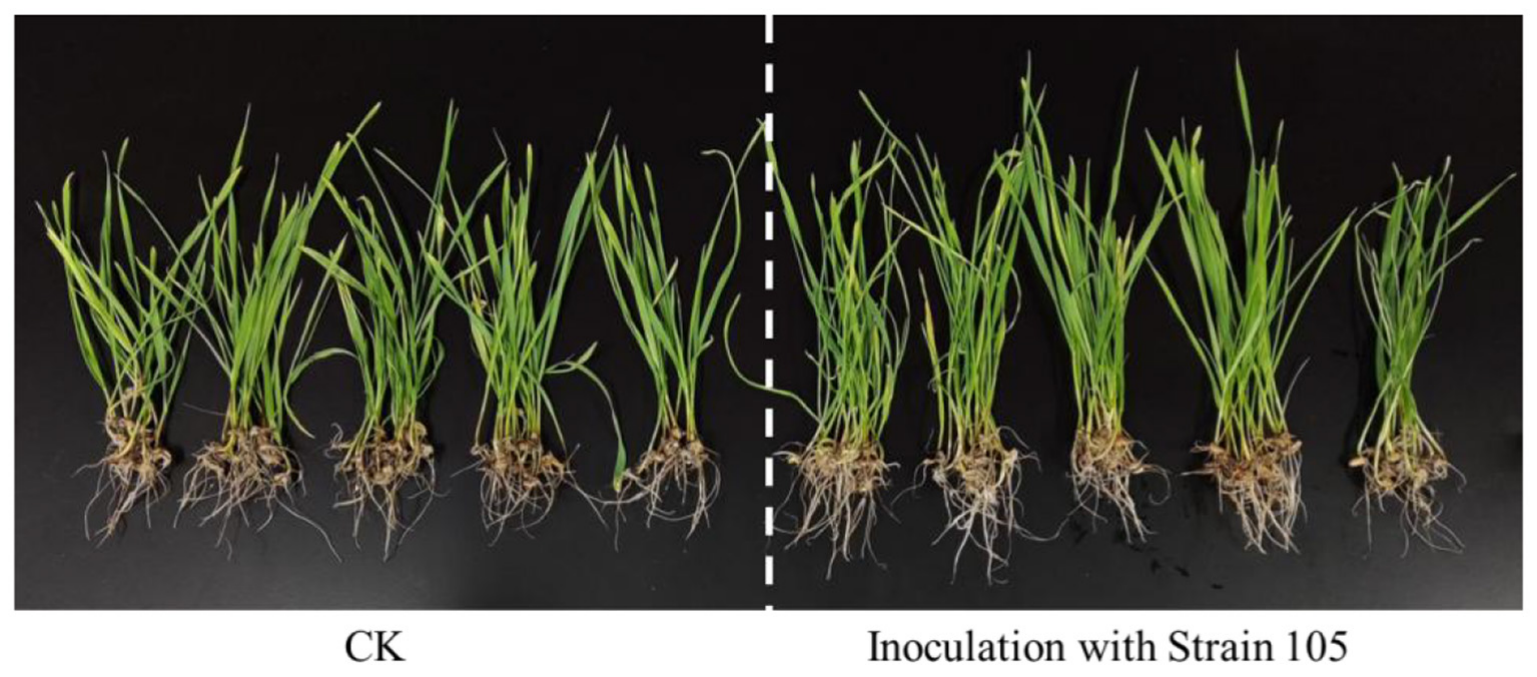
Growth-promoting effect of Strain 105 on wheat under 200 mmol/L NaCl stress.

In the hydroponic experiment under salt stress (200 mmol/L NaCl), the growth parameters of wheat seedlings in the Strain 105 treatment group were significantly superior to those in the control group (Figures 5, 6). After 14 days of co-cultivation, the root length of wheat in the treatment group reached 4.8 cm, representing a 20% increase compared with the control group; the plant height reached 14.2 cm, showing an 18% increase relative to the control group. Both growth indices exhibited statistically significant differences confirming that Strain 105 exerts a prominent growth-promoting effect on wheat under salt-stressed conditions.

### 3.3 Whole genome sequencing and analysis of Strain 105

#### 3.3.1 General genomic features

**Figure 7:**
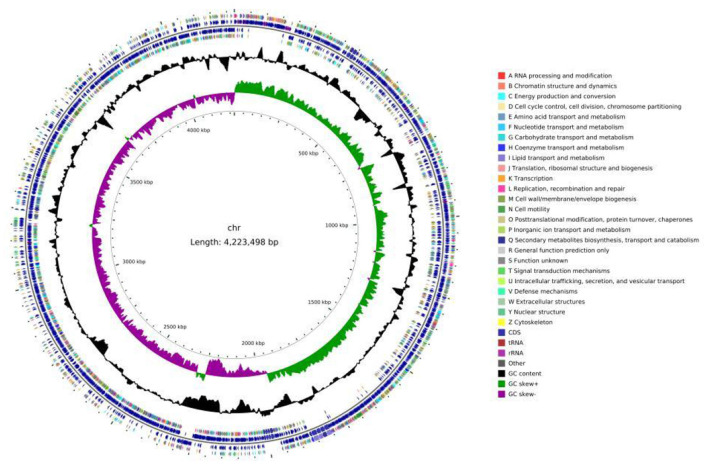
Genome circle map of Strain 105.

Whole genome sequencing of Strain 105 was performed using the Illumina NovaSeq (second-generation) and PacBio (third-generation) sequencing platforms. The Illumina NovaSeq platform generated a total of 6,432,614 reads, corresponding to a total sequence length of 971,324,714 bp. The PacBio platform yielded 178,990 reads with a cumulative length of 1,474,747,428 bp, and the longest read length reached 234,079 bp. The sequencing depth of the whole genome of Strain 105 was 228 × . After sequencing, the raw data were filtered, and all reads were assembled using Flye (v2.9.1-b1781). Finally, the genome of Strain 105 was determined to contain a single circular chromosome of 4,223,498 bp and a GC content of 43.55% (Figure 7). The complete genome sequence of Strain 105 was deposited in the NCBI GenBank database under accession number CP186921.

Genome annotation with PGAP predicted the circular chromosome to harbor a total of 4,380 genes, including 4,120 protein-coding genes, 30 rRNA genes, 86 tRNA genes, five ncRNA genes, and 139 pseudogenes. In addition, a total of 414 repetitive sequences were identified in the whole genome of Strain 105, which were classified into 22 short interspersed nuclear elements (SINEs), 81 long interspersed nuclear elements (LINEs), 197 long terminal repeats (LTRs), 93 transposons, and 13 repetitive sequences of unknown types.

#### 3.3.2 Genomic validation of taxonomic status

To clarify the taxonomic status of Strain 105, a comprehensive identification was performed by combining 16S rRNA sequence alignment, genome-wide ANI analysis, dDDH, and core genome phylogenetic tree construction.

**Figure 8:**
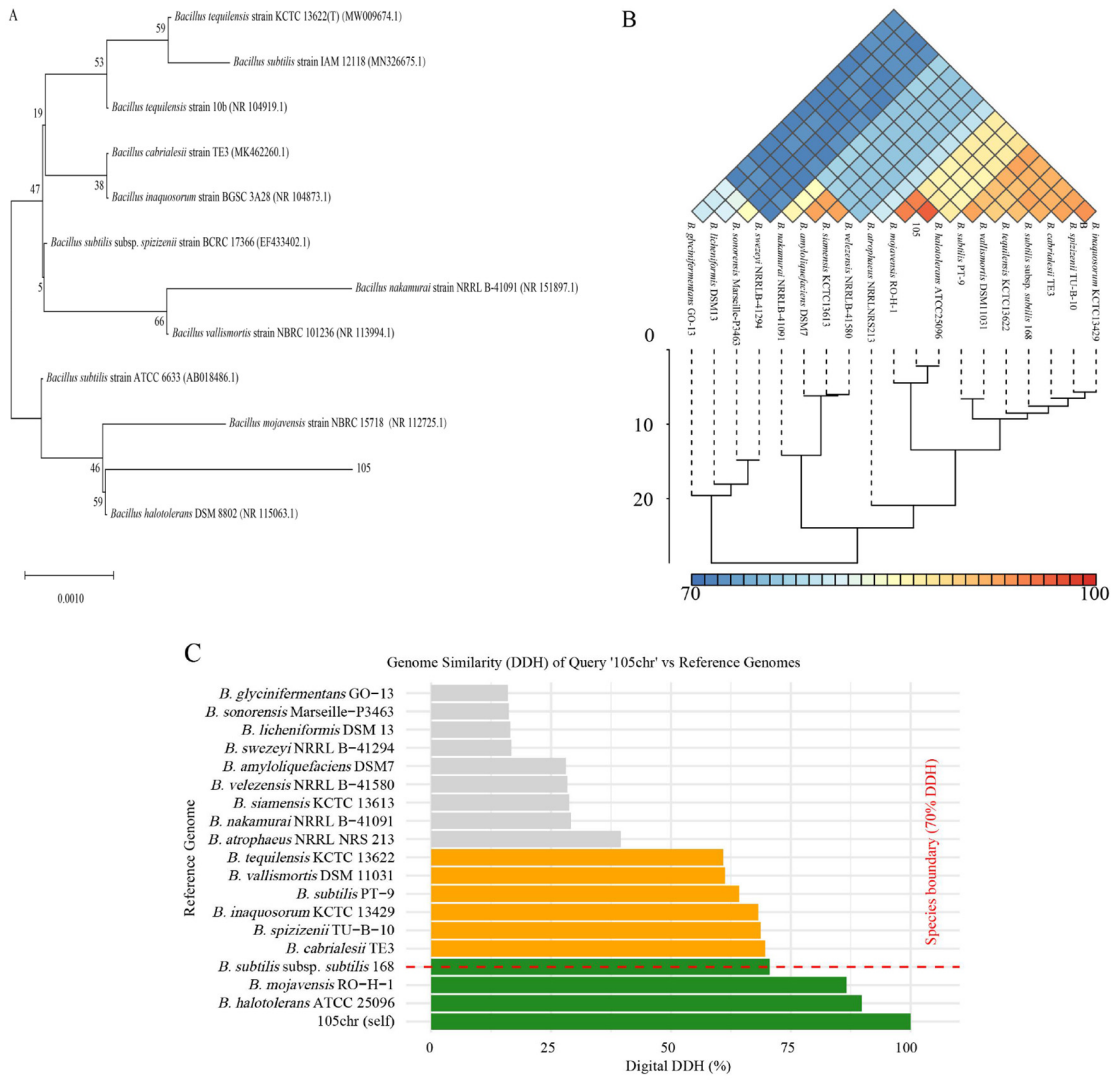
Taxonomic identification of Strain 105. **(A)** Phylogenetic analysis based on the 16S rRNA-encoding gene. **(B)** ANI analysis. **(C)** dDDH results of Strain 105.

The 16S rRNA-encoding sequence of Strain 105 was aligned against the NCBI database, and a phylogenetic tree was constructed using the neighbor-joining method with molecular evolutionary genetics analysis 5 (MEGA5) software. The results showed that this strain and *B. halotolerans* DSM 8802 clustered within the same minimum clade, exhibiting the closest evolutionary distance (Figure 8A).

ANI analysis was conducted between Strain 105 and 20 reference strains of the genus *Bacillu*s. The ANI values between Strain 105 and these reference strains ranged from 78.5 to 97.8%, showing significant differences in genetic similarity (Figure 8B). Among them, the ANI value between Strain 105 and *B. halotolerans* ATCC 25096 (GCF_001517105.1) was the highest, reaching 97.8%, which was significantly higher than the universally recognized ANI threshold (95%−96%) for bacterial species-level classification. This result was further supported by the dDDH analysis: the dDDH value between Strain 105 and *B. halotolerans* ATCC 25096 was 89.8%. Since this value is well above the 70% threshold for species delineation, it confirms that Strain 105 belongs to the species *Bacillus halotolerans* (Figure 8C).

Collectively, the morphological characteristics and multi-dimensional genomic analyses conclusively identified Strain 105 as *B. halotolerans*.

#### 3.3.3 Genome functional annotation

**Figure 9:**
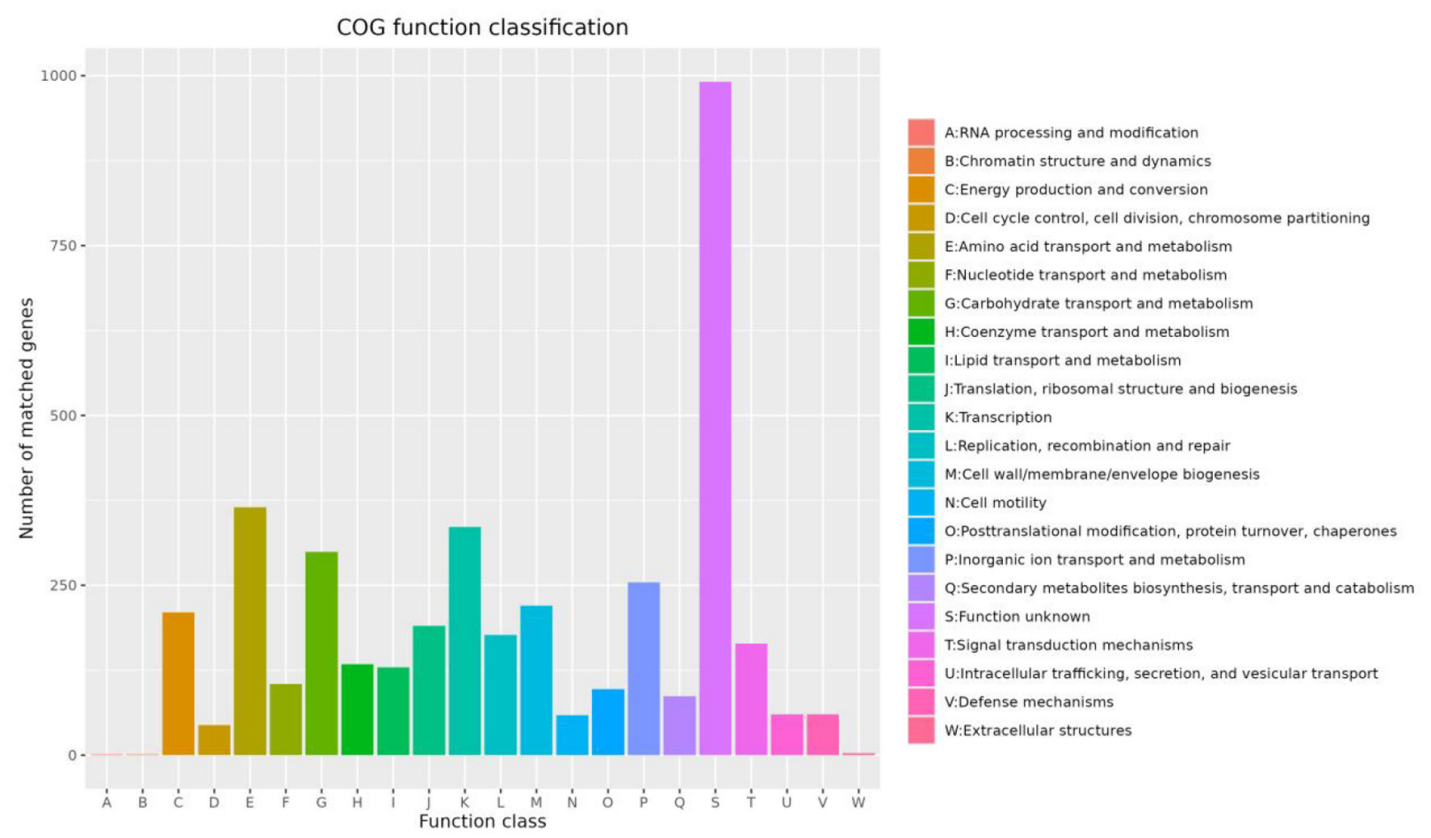
COG functional annotation of the Strain 105 genome.

Annotation against the COG database showed that a total of 3,986 genes in the whole genome of Strain 105 could be assigned to 22 functional categories (Figure 9). Among these genes, 2,995 were protein-coding genes with defined functional classifications. Specifically, the largest number of genes encoded functional proteins associated with amino acid transport and metabolism, accounting for 365 genes (9.2% of the annotated genes). This was followed by genes encoding transcription factors, with 336 genes (8.4%). In addition, 299 genes (7.5%) were involved in carbohydrate transport and metabolism. A total of 60 genes (1.5%) were related to defense mechanisms, providing a genetic basis for the observed antimicrobial activity of Strain 105. Furthermore, 991 genes were classified as genes with unknown functions, leaving room for further functional mining.

### 3.4 Gene clusters related to core biological functions

#### 3.4.1 Antimicrobial-associated gene modules

To elucidate the genetic basis underlying this broad-spectrum antagonistic activity, the whole genome of strain 105 was analyzed for biosynthetic gene clusters (BGCs) of secondary metabolites using antiSMASH. A total of 10 secondary metabolite gene clusters were identified in Strain 105 (Table 1). Among these, five clusters showed 100% similarity to known functional gene clusters, including a trans-AT PKS/NRPS cluster homologous to bacillaene (Region 4), an NRPS cluster matching fengycin (Region 5), an NRPS cluster corresponding to the siderophore bacillibactin (Region 8), a sactipeptide cluster identical to subtilosin A (Region 9), and a cluster encoding the dipeptide antibiotic bacilysin (Region 10). Bacillaene and bacilysin exhibit antagonistic activity against both bacteria and fungi, fengycin displays antifungal activity, whereas bacillibactin and subtilosin A possess antibacterial activity.

**Table 1:** Secondary metabolite gene clusters of strain 105.

Region	Type	Most similar known cluster	Similarity	Antimicrobial spectrum
Region 1	NRPS	Surfactin NRP: Lipopeptide	86%	
Region 2	Lanthipeptide-class-iii	-	-	
Region 3	Terpene	-	-	
Region 4	transat-PKS/NRPS	Bacillaene	100%	Antibacterial and antifungal
Region 5	NRPS	Fengycin	100%	Antifungal
Region 6	Terpene	-	-	
Region 7	T3PKS	Laterocidine	5%	
Region 8	NRPS	Bacillibactin	100%	Antibacterial
Region 9	Sactipeptide	Subtilosin A	100%	Antibacterial
Region 10	Dipeptide synthetase	Bacilysin	100%	Antibacterial and antifungal

In addition, multiple protease-encoding genes were identified in the genome (see Supplementary Table S1), including *prtG* (ACO9EM_00280), *htrA* (ACO9EM_07085), and *isp* (ACO9EM_07240). The expression products of these genes can degrade the cell wall components of pathogenic microorganisms. This coexistence of diverse antimicrobial BGCs and hydrolase-encoding genes provides a comprehensive genomic repertoire that correlates with the observed broad-spectrum antagonistic phenotype of Strain 105.

#### 3.4.2 Salt tolerance–associated gene modules

Genomic analysis identified genes involved in compatible solute (osmoprotectant) biosynthesis/transport and ion homeostasis, which are likely to underpin the extreme salt tolerance of Strain 105. The salt tolerance–associated genes of Strain 105 mediate adaptation to salt stress primarily through two major pathways: (1) osmoprotectant synthesis and transport and (2) ion balance regulation, which cover five functional categories (Table 2).

**Table 2:** Salt tolerance-associated genes in Strain 105.

Type	Gene	Locustag	Product
Betaine metabolism	*mmuM*	ACO9EM_01405	Homocysteine S-methyltransferase
	*opuAA*	ACO9EM_01725	Glycine/proline betaine ABC transporter ATP-binding protein OpuAA
	*opuAB*	ACO9EM_01730	Glycine/proline betaine ABC transporter permease subunit OpuAB
			ACO9EM_01735	Glycine betaine ABC transporter substrate-binding protein
			ACO9EM_09895	Choline esterase
	*opuD*	ACO9EM_15865	Glycine betaine transporter OpuD
	*gbsB*	ACO9EM_16435	Choline dehydrogenase
	*betB*	ACO9EM_16440	Betaine-aldehyde dehydrogenase
	*cudC*	ACO9EM_16445	Choline uptake/conversion transcriptional regulator CudC
	*opuBC*	ACO9EM_17830	Choline ABC transporter substrate-binding lipoprotein OpuBC
	*opuBA*	ACO9EM_17840	Choline ABC transporter ATP-binding protein OpuBA
	*opuCD*	ACO9EM_1787	Glycine betaine/carnitine/choline/choline sulfate ABC transporter permease OpuCD
	*opuCC*	ACO9EM_17880	Osmoprotectant ABC transporter substrate-binding lipoprotein OpuCC
*opuCB*	ACO9EM_17885	Glycine betaine/carnitine/choline/choline sulfate ABC transporter permease OpuCB
*opuCA*	ACO9EM_17890	Osmoprotectant ABC transporter ATP-binding protein OpuCA
Proline metabolism		ACO9EM_05575	YhgE/Pip family protein
	*proG*	ACO9EM_07090	Pyrroline-5-carboxylate reductase ProG
	*proB*	ACO9EM_07195	Glutamate 5-kinase
	*proA*	ACO9EM_07200	Glutamate-5-semialdehyde dehydrogenase
	*proC*	ACO9EM_10080	Pyrroline-5-carboxylate reductase
	*proC*	ACO9EM_12890	Pyrroline-5-carboxylate reductase
	*comER*	ACO9EM_13825	Late competence protein ComER
	*rocD*	ACO9EM_21380	Ornithine aminotransferase
Ectoine metabolism		ACO9EM_02160	Aspartate kinase
	*asd*	ACO9EM_09065	Aspartate-semialdehyde dehydrogenase
	*dapG*	ACO9EM_09070	Aspartate kinase
		ACO9EM_15045	Aspartate kinase
Trehalose metabolism	*treP*	ACO9EM_04200	PTS system trehalose-specific EIIBC component
	*treC*	ACO9EM_04205	α,α-phosphotrehalase
	*treR*	ACO9EM_04210	Trehalose operon repressor
	*glvA*	ACO9EM_04465	Maltose6′-phosphate glucosidase
	*malP*	ACO9EM_04475	PTS maltose transporter subunit IIBC
			ACO9EM_21790	Maltose acetyltransferase domain-containing protein
Ktr system (ion homeostasis)	*ktrD*	ACO9EM_07400	Ktr system potassium transporter KtrD
	*ktrC*	ACO9EM_07925	Ktr system potassium transporter KtrC
Trk system (ion homeostasis)	*trkH*	ACO9EM_16460	TrkH family potassium uptake protein

The compatible solutes mainly include betaine, proline, ectoine, and trehalose. Among these, the betaine synthesis and transport system is the most complete, comprising biosynthesis genes such as choline dehydrogenase (*gbsB*, ACO9EM_16435) and betaine-aldehyde dehydrogenase (*betB*, ACO9EM_16440), as well as transport-related genes, including those of the ABC transporter family (*opuAA*, ACO9EM_01725; *opuAB*, ACO9EM_01730; *opuBC*, ACO9EM_17830; *opuCA*, ACO9EM_17890) and the specific transporter *opuD* (ACO9EM_15865). In addition, the strain harbors the choline uptake regulator gene *cudC* (ACO9EM_16445) and a cholinesterase gene (ACO9EM_09895), which can ensure the supply of precursor substances.

The key genes involved in proline synthesis are complete, including glutamate 5-kinase (*proB*, ACO9EM_07195), glutamate-5-semialdehyde dehydrogenase (*proA*, ACO9EM_07200), and pyrroline-5-carboxylate reductase (*proC/G*; ACO9EM_10080, ACO9EM_12890, ACO9EM_07090). Additionally, ornithine aminotransferase (*rocD*, ACO9EM_21380) was identified, which can mediate the supplementation of proline precursors.

Genes present that are related to ectoine synthesis included aspartokinase (ACO9EM_02160, ACO9EM_09070, ACO9EM_15045) and aspartate-semialdehyde dehydrogenase (*asd*; ACO9EM_09065). Trehalose metabolism relies on the PTS system-specific component *treP* (ACO9EM_04200), α,α-phosphotrehalase (*treC*, ACO9EM_04205), and the operon repressor *treR* (ACO9EM_04210).

Ion balance regulation involves the Ktr system (*ktrC*, ACO9EM_07925; *ktrD*, ACO9EM_07400) and the Trk system (TrkH family protein, ACO9EM_16460), which maintain intracellular ion homeostasis via selective potassium uptake. This analysis indicates that the coordinated expression of the above genes enables Strain 105 to resist high-salt stress through multiple osmoprotective mechanisms and sustain normal cellular physiological functions.

#### 3.4.3 Growth promotion–associated gene modules

PGPR can directly facilitate plant growth via the biosynthesis of auxins. In bacteria, multiple tryptophan-dependent pathways exist for the synthesis of IAA. Among these pathways, the indole pyruvic acid (IPyA) pathway is the most prevalent.

Based on the previously reported IPyA pathway, genome-wide alignment was performed to identify the genes involved in this pathway in Strain 105, such as *trpE/D/B* and *ipdC*. As presented in Table 3, in Strain 105, chorismate is first converted to anthranilate by anthranilate synthase (TrpE). Subsequently, anthranilate is transformed into indole-3-glycerol phosphate by anthranilate phosphoribosyltransferase (TrpD), which is further converted to tryptophan under the catalysis of tryptophan synthase (TrpB). Tryptophan is then converted to indole pyruvic acid by tryptophan aminotransferase, followed by the conversion of IPyA to indole-3-acetaldehyde by IlvB and IlvN. Finally, indole-3-acetaldehyde is synthesized into IAA under the action of the gene products encoded by ACO9EM_20125 and ACO9EM_21140. Therefore, Strain 105 synthesizes auxin through the pathway of tryptophan → indole pyruvic acid → indole-3-acetaldehyde → IAA, which is consistent with the IAA yield detected in the phenotypic assay.

**Table 3:** IAA synthesis-related genes in Strain 105.

Gene	Locustag	Product
*trpE*	ACO9EM_12325	Anthranilate synthase component I
	ACO9EM_00445	Anthranilate synthase component I family protein
*pabA*	ACO9EM_00450	Aminodeoxychorismate/anthranilate synthase component II
*trpD*	ACO9EM_12320	Anthranilate phosphoribosyltransferase
*trpB*	ACO9EM_12305	Tryptophan synthase subunit beta
*trpA*	ACO9EM_12300	Tryptophan synthase subunit alpha
*trpC*	ACO9EM_12315	Indole-3-glycerol phosphate synthase TrpC
*ilvN*	ACO9EM_14955	Acetolactate synthase small subunit
*ilvB*	ACO9EM_14960	Acetolactate synthase large subunit
*alsS*	ACO9EM_19035	Acetolactate synthase AlsS
*dhaS*	ACO9EM_10590	Aldehyde dehydrogenase DhaS
*betB*	ACO9EM_16440	Betaine-aldehyde dehydrogenase
	ACO9EM_20125	Aldehyde dehydrogenase
	ACO9EM_21140	Aldehyde dehydrogenase family protein

In addition, genes encoding high-activity proteases (e.g., *htrA* and *isp*) in Strain 105 can promote the mineralization of soil organic nitrogen, providing available nutrients for plants and thereby indirectly enhancing the growth-promoting effect.

#### 3.4.4 Rhizosphere colonization–associated gene modules

Motility and chemotaxis are crucial factors influencing the rhizosphere colonization ability of microorganisms. A complete chemotactic pathway was identified in Strain 105 (see Supplementary Table S2). The genome of Strain 105 contains 10 genes encoding methyl-accepting chemotaxis proteins (MCPs) that perceive external signals, as well as genes involved in chemotactic signal transduction: *cheA* (ACO9EM_08905) encoding a histidine kinase, *cheW* (ACO9EM_08910) encoding a scaffold coupling protein, *cheY* (ACO9EM_08855) encoding a stress response regulator protein, *cheR* (ACO9EM_12345) encoding a methyltransferase, and *cheB* (ACO9EM_08900) encoding a methylesterase.

PGPR can form biofilms on plant roots, thereby enhancing their rhizosphere colonization capacity. In Strain 105, a set of genes covering the entire regulatory cycle and structural assembly of biofilm formation were identified, involving multiple functional modules such as transcriptional regulation, signal transduction, matrix synthesis, and surface modification (see Supplementary Table S3). At the transcriptional regulation level, the identified genes include those of the *abrB* family (ACO9EM_00250, ACO9EM_05795), the s*inR/sinI* regulatory pair (ACO9EM_13315, ACO9EM_13310), the GTP-sensing regulator CodY (ACO9EM_08770), and the sporulation transcription factor Spo0A (ACO9EM_13120). Among these, members of the AbrB family possess both transition-phase gene transcriptional regulation and anti-repression functions (e.g., *abbA*, ACO9EM_07730), and SinI can relieve the inhibition of biofilm formation by antagonizing the activity of SinR. The signal transduction system is dominated by two-component systems, including histidine kinases KinC (ACO9EM_07915), DegS (ACO9EM_18745), and DesK (ACO9EM_10535), which mediate extracellular signal perception or the stress response. Notably, KinC can amplify signal transduction through the KinB pathway-activated protein KbaA (ACO9EM_00935).

Genes identified that are related to biofilm matrix and structural components include fibrin (TasA, ACO9EM_13320) and its anchor protein TapA (ACO9EM_13330), extracellular polysaccharide synthesis proteins (EpsB, ACO9EM_18200; EpsG, ACO9EM_18175; EpsE, ACO9EM_18185), and surface hydrophobic proteins BslA/B (ACO9EM_16450, ACO9EM_20045). In addition, extracellular matrix-specific regulatory factors RemA/B (ACO9EM_08520, ACO9EM_00025), the biofilm formation stimulator Veg (ACO9EM_00285), and biofilm-specific peroxidase AhpA (ACO9EM_07780) were also identified in Strain 105.

The coordinated action of the above-mentioned genes constitutes a complete regulatory network for biofilm formation in Strain 105, providing a molecular basis for the initial attachment, matrix accumulation, and structural stabilization of biofilms.

## 4 Discussion

**Figure 10:**
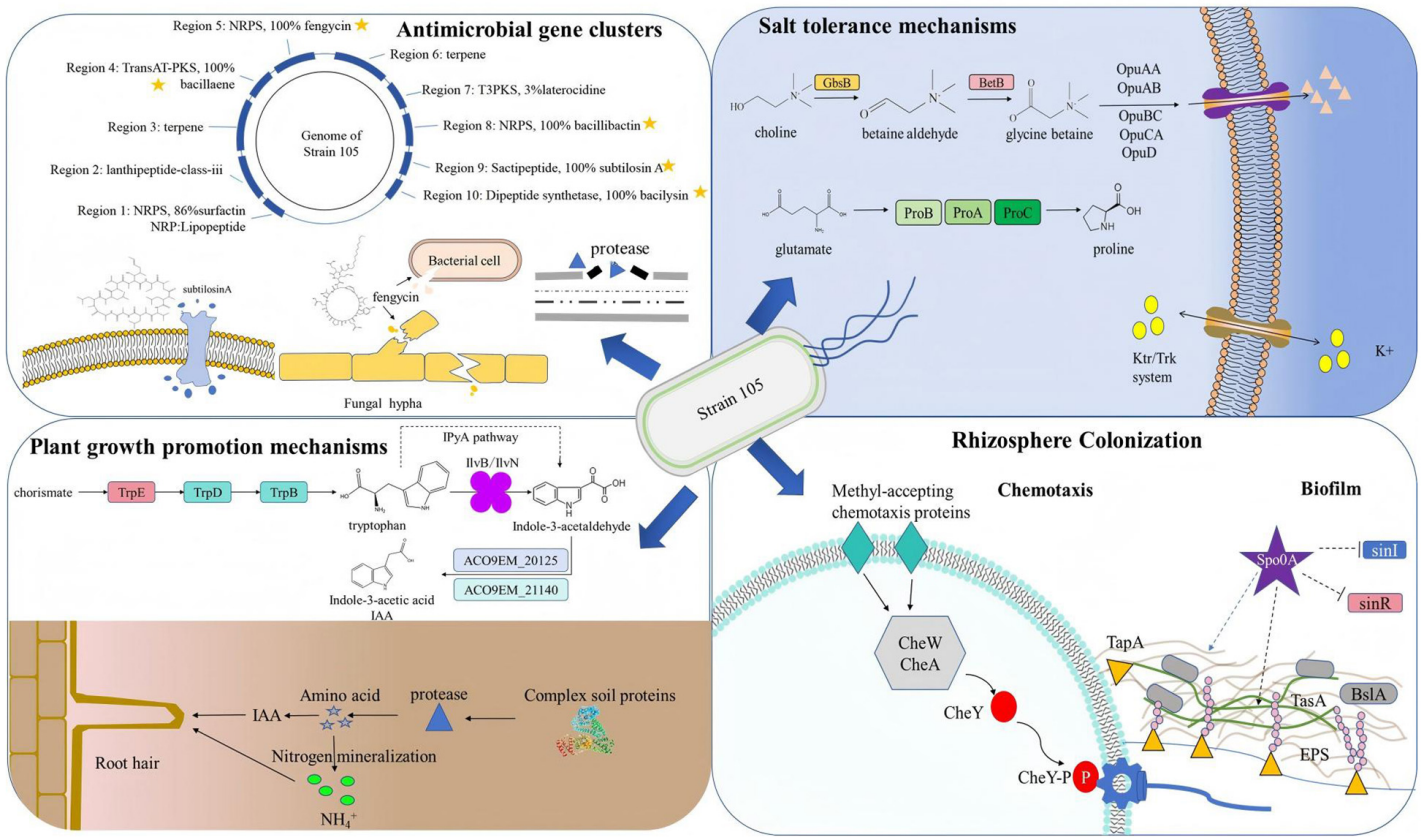
Growth-promoting mechanism of Strain 105.

To integrate the phenotypic, physiological, and genomic findings of this study, we present a unified mechanistic model that elucidates how *Bacillus halotolerans* Strain 105 functions as a multifunctional PGPR capable of concurrently mitigating soil salinity stress and phytopathogen pressure—two pivotal challenges in sustainable agriculture. The integrative schematic in Figure 10 highlights the following four tightly interconnected functional modules that collectively underpin the ecological fitness and agronomic potential of Strain 105:

(i) extreme salt tolerance, mediated by a comprehensive osmoprotective system—including uptake of exogenous glycine betaine via Opu transporters, *de novo* synthesis of compatible solutes through the *gbsAB* and *betAB* pathways, efficient K^+^/Na^+^ homeostasis (*kdp, trk* systems), and cell envelope stabilization—enabling active growth at up to 14% NaCl, which is among the highest salinity thresholds reported for *Bacillus* species to date;
(ii) phytohormone-driven growth promotion, primarily through IAA biosynthesis via the indole-3-pyruvate pathway (*ipdC*-dependent), which modulates the root system architecture and enhances nutrient acquisition in wheat seedlings even under 200 mmol/L NaCl stress;
(iii) broad-spectrum antimicrobial activity, conferred by a rich repertoire of secondary metabolite BGCs, including those encoding iturin, fengycin, bacillibactin, and bacilysin, whose products directly inhibit diverse fungal (e.g., *Fusarium oxysporum, F. graminearum*) and bacterial phytopathogens;
(iv) effective rhizosphere colonization, supported by a suite of genetic determinants governing chemotaxis toward root exudates (*che* genes), biofilm formation (*eps, tasA* operons), and surface adhesion—ensuring persistent root association necessary for the sustained delivery of growth-promoting and protective functions.

Critically, these four modules operate synergistically: salt tolerance enables survival in saline soils; successful colonization establishes a stable rhizosphere niche; and within this niche, the continuous production of IAA and antimicrobial metabolites directly benefits the host plant. This functional integration positions Strain 105 as a promising candidate for next-generation bioinoculants tailored for marginal, pathogen-prone agricultural environments. To further highlight this potential, we here discuss each module in the context of current literature, evaluate their interdependencies, and outline translational pathways toward field application.

The salt tolerance of Strain 105 is substantially superior to that of most previously reported *Bacillus* strains. The maximum salt tolerance limit of *B. pumilus* strain JPVS11 is 11% NaCl (Kumar et al., 2021) and the halotolerant strain *B. halotolerans* KKD1 can tolerate up to 13% NaCl (Wu et al., 2022). In contrast, Strain 105 maintained vigorous reproductive capacity even under 14% NaCl conditions. This characteristic of Strain 105 benefits from the dual mechanism of the *de novo* synthesis and high-affinity uptake of compatible solutes. The presence of *gbsB* and *betB* in the genome of Strain 105 enables it to synthesize glycine betaine using choline as a substrate, a pathway that has been confirmed as a key mechanism for *B. subtilis* to adapt to salt stress (Boch et al., 1997; Nau-Wagner et al., 2012). The genome of Strain 105 also contains multiple genes encoding Opu transporters (*opuAA-CA, opuD*). These transporters can efficiently uptake exogenous betaine from the rhizosphere environment, thereby enhancing the adaptability of the strain for rhizosphere colonization in environments with fluctuating salt concentrations (Kappes et al., 1996). In addition to the glycine betaine pathway, the complete proline synthesis operon (*proB-proC*) in the genome leads to the massive accumulation of proline, which can significantly enhance the cell membrane stability of Strain 105 under 200 mmol/L NaCl stress. This result is consistent with the findings in *Halomonas elongata* OUT30018 (Muñoz-Torres et al., 2024). Such multiple salt tolerance mechanisms would endow Strain 105 with a competitive advantage in saline-alkali soils.

Another notable characteristic of Strain 105 is its broad-spectrum antibacterial activity, inhibiting seven plant pathogenic fungi, including four *Fusarium* species that cause significant global crop yield losses (Dean et al., 2012). AntiSMASH analysis of the genome revealed that Strain 105 contains five complete known antibiotic BGCs, responsible for the synthesis of bacillaene, bacilysin, fengycin, bacillibactin, and subtilosin A, respectively. These metabolites construct a multi-target defense barrier through synergistic effects. Fengycin disrupts the phospholipid bilayer of the pathogen's cell membrane, causing leakage of cellular contents and ultimately leading to cell death (Yin et al., 2024). Bacillaene and bacillibactin can inhibit prokaryotic protein synthesis (Patel et al., 1995), while subtilosin A interferes with proteins and RNA (Babasaki et al., 1985). This multifunctionality also minimizes the evolutionary risk of pathogen resistance. In addition, 26 protease-encoding genes annotated in the strain's genome enable Strain 105 to inhibit mycelial growth by secreting proteases, a mechanism that has been verified in *Trichoderma harzianum* to inhibit *F. oxysporum* (Liu and Yang, 2007).

This study found that Strain 105 can significantly increase the root length and plant height of wheat under salt stress (increased by 20% and 18%, respectively). This growth-promoting effect may stem from the synergistic action of multiple mechanisms. First, genomic analysis confirmed that Strain 105 possesses a complete IAA synthesis pathway (the IPyA pathway), and 13.6 μg/mL of IAA was detected in its fermentation broth. As a key growth hormone, IAA can directly stimulate plant cell division and elongation, and this hormone is particularly helpful for maintaining root development under stress conditions (Jacob et al., 2025). Second, the strain's protease-producing activity (216.42 IU) may help to mineralize soil organic nitrogen into plant-available forms, thereby indirectly promoting nutrient absorption (Sieradzki et al., 2023). Of note, the growth-promoting effect of Strain 105 was observed in a salt-stressed hydroponic system. This indicates that the functions of Strain 105 are not isolated; that is, the IAA and enzymes produced may play a fundamental role in regulating the overall physiological state of plants and enhancing their stress resilience. This is consistent with the recent proposal that PGPR cope with complex stresses by “priming” plant immunity and metabolism (Mhlongo et al., 2021).

Efficient rhizosphere colonization ability is a prerequisite for PGPR strains to exert sustained effects. The genome of Strain 105 contains complete chemotaxis and motility gene modules, including motility-related genes (*motA/B, fli* family genes) and chemotaxis regulatory genes (the *che* operon and 10 genes encoding MCPs). These genes enable Strain 105 to perceive and actively move toward chemical substances secreted by plant roots (such as organic acids and carbohydrates). Additionally, Strain 105 harbors genes involved in biofilm formation (*tasA, eps, bslA/B*) and a complex regulatory network, including key transcriptional regulators (*sinR, spo0A*), signal perception systems (*kinC*), and matrix synthesis genes (*tasA, eps* operon). The biofilm formation process regulated by the *sinR/sinI* and *spo0A* genes would allow the strain to firmly attach to plant roots and resist osmotic stress, as confirmed in the colonization of tomatoes and Arabidopsis by *B. subtilis* NCIB 3610 (Pomerleau et al., 2024). As a core factor regulating sporulation, biofilm development, and stress response, the presence of the *spo0A* gene suggests that Strain 105 can dynamically switch its lifestyle according to rhizosphere environmental signals, thereby maximizing its ecological adaptability. These robust chemotaxis, motility, and biofilm-forming capabilities collectively constitute the genetic basis for Strain 105 to efficiently colonize the plant rhizosphere and establish a mutually beneficial niche.

This study has certain limitations that should be acknowledged. First, all plant experiments were completed under controlled hydroponic conditions. Field trials in actual salinized farmland ecosystems are required to evaluate the practical application effect of the strain in complex microbial communities. Second, although the genomic evidence provides strong support for the functional characteristics of the strain, gene-knockout experiments (such as knocking out *gbsB* or the fengycin synthesis gene cluster) are still needed to further clarify the causal relationship between genotype and phenotype.

In summary, *B. halotolerans* 105 is a multifunctional PGPR strain with broad-spectrum antibacterial activity, high salt tolerance, and significant growth-promoting ability. Its complete genomic information reveals the complex genetic network of synergistic effects underlying these phenotypes. This study not only provides a promising candidate strain for the microbial remediation of saline-alkali soil agriculture but also, more importantly, offers a research paradigm for understanding the versatility of PGPR from a systems biology perspective. Future work should focus on the functional verification of key genes and the evaluation of application value in the field to promote the practical use of Strain 105 in the development of green and sustainable agriculture.

## Data Availability

The datasets presented in this study can be found in online repositories. The names of the repository/repositories and accession number(s) can be found in the article/Supplementary material. The complete genome sequence of *B. halotolerans* 105 has been deposited in GenBank under the accession number CP186921.
